# KDIGO-based acute kidney injury criteria operate differently in hospitals and the community—findings from a large population cohort

**DOI:** 10.1093/ndt/gfw052

**Published:** 2016-04-07

**Authors:** Simon Sawhney, Nick Fluck, Simon D. Fraser, Angharad Marks, Gordon J. Prescott, Paul J. Roderick, Corri Black

**Affiliations:** 1 Institute of Applied Health Sciences, University of Aberdeen, Aberdeen, UK; 2 Renal Unit, NHS Grampian, Aberdeen, UK; 3 Faculty of Medicine, University of Southampton, Southampton, UK

**Keywords:** acute kidney injury, delivery of health care, epidemiology, primary health care, survival analysis

## Abstract

**Background:**

Early recognition of acute kidney injury (AKI) is important. It frequently develops first in the community. KDIGO-based AKI e-alert criteria may help clinicians recognize AKI in hospitals, but their suitability for application in the community is unknown.

**Methods:**

In a large renal cohort (*n* = 50 835) in one UK health authority, we applied the NHS England AKI ‘e-alert’ criteria to identify and follow three AKI groups: hospital-acquired AKI (HA-AKI), community-acquired AKI admitted to hospital within 7 days (CAA-AKI) and community-acquired AKI not admitted within 7 days (CANA-AKI). We assessed how AKI criteria operated in each group, based on prior blood tests (number and time lag). We compared 30-day, 1- and 5-year mortality, 90-day renal recovery and chronic renal replacement therapy (RRT).

**Results:**

In total, 4550 patients met AKI e-alert criteria, 61.1% (2779/4550) with HA-AKI, 22.9% (1042/4550) with CAA-AKI and 16.0% (729/4550) with CANA-AKI. The median number of days since last blood test differed between groups (1, 52 and 69 days, respectively). Thirty-day mortality was similar for HA-AKI and CAA-AKI, but significantly lower for CANA-AKI (24.2, 20.2 and 2.6%, respectively). Five-year mortality was high in all groups, but followed a similar pattern (67.1, 64.7 and 46.2%). Differences in 5-year mortality among those not admitted could be explained by adjusting for comorbidities and restricting to 30-day survivors (hazard ratio 0.91, 95% confidence interval 0.80–1.04, versus hospital AKI). Those with CANA-AKI (versus CAA-AKI) had greater non-recovery at 90 days (11.8 versus 3.5%, P < 0.001) and chronic RRT at 5 years (3.7 versus 1.2%, P < 0.001).

**Conclusions:**

KDIGO-based AKI criteria operate differently in hospitals and in the community. Some patients may not require immediate admission but are at substantial risk of a poor long-term outcome.

## INTRODUCTION

Acute kidney injury (AKI) is common, serious and under-recognized [1, 2]. The Kidney Disease: Improving Global Outcomes (KDIGO) creatinine change criteria are typically used for diagnosis [3]. There is a need to recognize AKI early to minimize preventable harm [4]. This has led to growing international interest in AKI ‘e-alerts’, based on KDIGO criteria, to warn clinicians when serum creatinine is rising abruptly in hospital patients [5–8]. However, an AKI episode is often already established before a patient reaches hospital [6, 9, 10]. AKI e-alerts are therefore also being considered for the community [5].

The KDIGO criteria define AKI based on changes in creatinine that are ‘presumed to have occurred within the prior 7 days’ [3]. Not all patients have had tests within a week, and this has led to pragmatic adaptations when operationalizing the KDIGO criteria in clinical practice (e-alerts) and in clinical research [11]. For example, AKI e-alerts developed by NHS England use three different look-back periods to accommodate this difficulty [5]. We have previously shown that these adaptations involve a ‘trade-off’ between identifying all clinically relevant AKI patients and misclassifying patients who do not have AKI [11, 12]. Importantly, this trade-off may not be the same for patients in the community who do not receive blood tests with the same regularity. This has consequences for whether the same criteria can be used to detect relevant AKI patients both in and out of hospital.

While it is acknowledged that AKI frequently emerges first in the community, this is based on hospital-only studies that identified patients who already had an elevated serum creatinine at the time of hospital admission [6, 9, 10]. These studies do not describe AKI occurring in the community that has not led to hospital admission. Hospital-only studies also cannot confirm whether the same criteria perform consistently in all clinical settings or remain clinically relevant without the context of a hospital admission. While one previous study has focussed on AKI in the community without hospital admission, it did not use a conventional application of the KDIGO criteria and did not provide a comparison with patients who were subsequently admitted or already in hospital [13].

Grampian Laboratory Outcomes Morbidity and Mortality Study-II (GLOMMS-II) is a population cohort linking national and regional data sources in a single UK health authority (adult population 438 332 [14]). It has been extensively used in renal research, including in the study of AKI definitions, and has been described in detail previously [11, 15–17]. Uniquely, all biochemistry is provided by a single biochemistry service, regardless of clinical location (inpatient, outpatient, community), ensuring that AKI in both primary and secondary care has been appropriately represented. Data linkage enables follow-up without formal patient recruitment, which minimizes any selection biases.

The aim of this study was to understand whether AKI e-alert criteria used in hospitals have similar implications when applied in the community, including those later admitted and not admitted. We used GLOMMS-II to describe three AKI groups: hospital-acquired AKI (HA-AKI), community-acquired AKI patients who were admitted to hospital within 7 days (CAA-AKI) and community-acquired AKI not admitted within 7 days (CANA-AKI). We described the characteristics of patients in each group, explored how the AKI e-alert criteria performed and compared their short- and long-term outcomes.

## MATERIALS AND METHODS

### Population

GLOMMS-II is a population cohort developed through novel data linkage of regional biochemistry to hospital episode data and the Scottish Renal Registry (SRR) for chronic renal replacement therapy (RRT), morbidity and outcomes [11, 16]. Information Services Division (ISD) Scotland refreshed these linkages using the community health index (CHI), a unique identifier for all residents in Scotland, to connect the timing of each AKI episode to individual hospital admissions. There were no patients without a CHI indexed in the ISD population ‘spine’, meaning that all records were linkable. ISD reports a precision of 99.9% for record linkages [18]. Use of GLOMMS-II was approved by regional research ethics and privacy advisory committees, and managed in Grampian Data Safe Haven [19].

**FIGURE 1:**
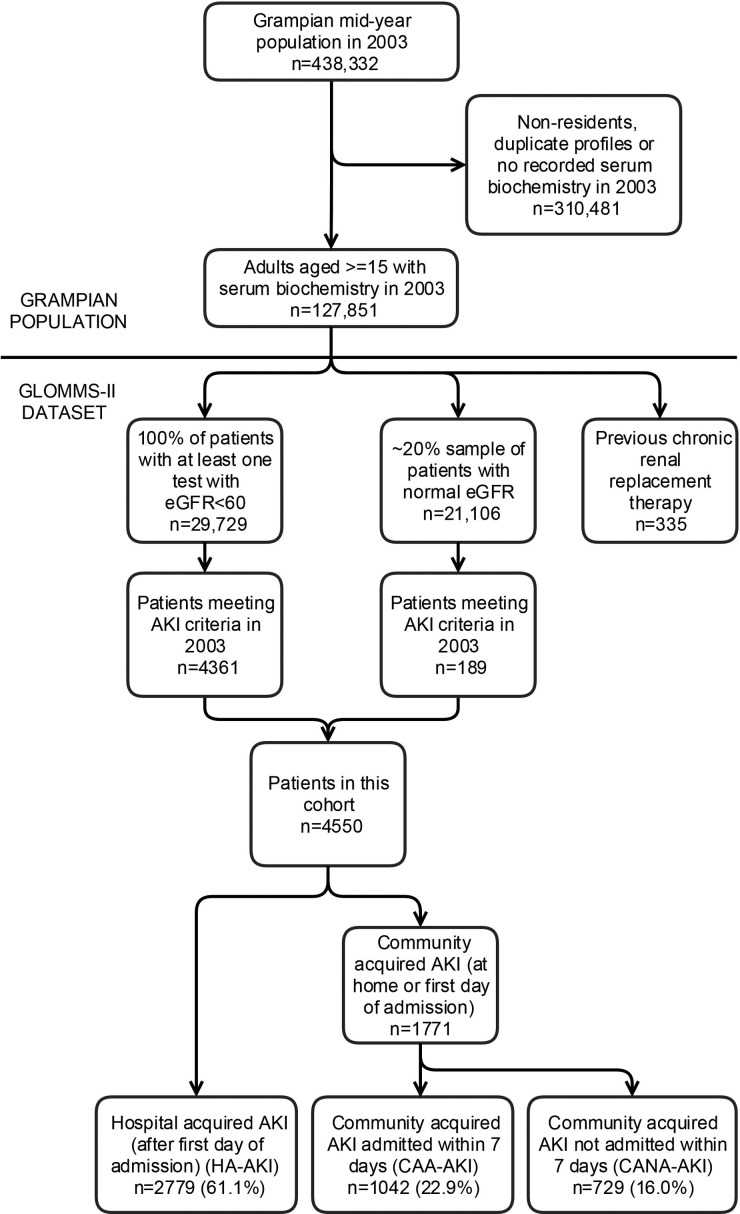
Flowchart of GLOMMS II cohort including AKI subgroups. AKI, acute kidney injury; HA-AKI, hospital AKI; CAA-AKI, community AKI admitted within 7 days; CANA-AKI, community AKI not admitted within 7 days. In sensitivity analysis, the proportions after multiplying out the sampled fraction were 61.0% HA-AKI, 22.1% CAA-AKI and 16.8% CANA-AKI.

GLOMMS-II includes 50 835 patients, 29 729 with at least one abnormal (estimated glomerular filtration rate, eGFR <60 mL/min/1.73 m^2^) result in 2003, and a ∼20% sample (21 106) of all those with normal results in 2003, with biochemistry from 1999 to 2009 for baseline and follow-up (Figure 1). A single biochemistry service processed all blood samples. All serum creatinines were isotope dilution mass spectrometry (IDMS) aligned. Patients with prior chronic RRT were excluded from this analysis.

### Exposure—hospital-acquired and community-acquired AKI

We applied the NHS England AKI e-alert Criteria 1–3 (Table 1) to identify AKI in 2003. We defined the first blood result meeting AKI criteria as the start of an ‘AKI episode’, and the corresponding value of the prior ‘look-back’ test was used as the ‘baseline’. From this baseline, we assigned AKI severity Stages 1–3 at initial diagnosis, and at peak severity using the highest creatinine within 90 days of AKI onset (Table 1). We have previously reported the application and good diagnostic performance of this algorithm elsewhere [11, 12].

**Table 1.** Description of AKI criteria, AKI group allocation and 90-day recovery status

Definition	Description
AKI criteria (1 of 3 required)	
Criterion 1	Serum creatinine ≥1.5 times higher than the median of all creatinine values 8–365 days ago
Criterion 2	Serum creatinine ≥1.5 times higher than the lowest creatinine within 7 days
Criterion 3	Serum creatinine >26 µmol/L higher than the lowest creatinine within 48 h
AKI severity stage	
Stage 1	Rise in creatinine of >26 µmol/L or index/baseline ≥1.5 and <2
Stage 2	Index/baseline ≥2 and <3
Stage 3	Index/baseline ≥3; or ≥1.5 and index creatinine >354 µmol/L (or 3 times the upper reference interval if age <18 years)
AKI group	
HA-AKI	Patient in hospital for >1 day when AKI developed
CAA-AKI	AKI presenting in the community and admitted within 7 days or presenting on the first day of hospital admission
CANA-AKI	AKI presenting in the community. Patient not subsequently admitted within 7 days
90-day recovery status	
Complete recovery	Latest creatinine within 90 days of AKI <1.2 times higher than the baseline creatinine
Partial recovery	Latest creatinine within 90 days of AKI <1.5 and ≥1.2 times higher than the baseline creatinine
Non-recovery	Lowest creatinine within 90 days of AKI ≥1.5 times higher than the baseline creatinine
‘Untested’	No repeat blood tests taken within 90 days of AKI diagnosis

AKI, acute kidney injury; HA-AKI, hospital AKI; CAA-AKI, community AKI admitted within 7 days; CANA-AKI, community AKI not admitted within 7 days.

We compared three AKI groups (Table 1): HA-AKI, CAA-AKI and CANA-AKI. We categorized AKI groups by linking the first AKI episode in 2003 to the closest hospital admission. AKI was ‘hospital-acquired’ if it developed in patients already in hospital (≥1 day) (HA-AKI). AKI was ‘community-acquired’ if it first developed in the community or on the first day of hospital admission ([6, 20]). We subcategorized community-acquired AKI into those subsequently admitted to hospital within 7 days (CAA-AKI) and those not admitted within 7 days (CANA-AKI).

Based on previous work, we recognized that some patients with smaller creatinine rises might have misclassified chronic kidney disease (CKD) [12], and that this might vary between AKI groups. Therefore, we also provided an alternative definition for AKI by restricting the AKI criteria to those who also had a doubling of serum creatinine during the AKI episode. By excluding patients with mild creatinine rises, this alternative definition misclassifies fewer patients with CKD [12].

### Outcomes

AKI patients were followed for up to 5 years from the date of first AKI. We described the operational performance of AKI criteria by reporting which AKI criteria were met on initial diagnosis, the number of days since last prior blood test and the number of blood tests in the prior year. We also reported initial and peak AKI severity stage for each AKI group and the number of patients meeting AKI criteria who also had a doubling of serum creatinine. We recorded mortality (from the national death registry) at 30 days, and 1 and 5 years after AKI onset. We determined renal recovery at 90 days, comparing the last creatinine within 90 days of AKI with the baseline creatinine (Table 1). We assessed whether patients had any repeat tests within 7 and 90 days of AKI onset. Finally, we recorded chronic RRT at 1 and 5 years. We defined chronic RRT using UK renal registry and SRR criteria [21].

### Covariates of interest

We reported age, sex, level of deprivation (most deprived quintile of the Scottish Index of Multiple Deprivation, SIMD) and rural location (settlement of <3000 people) [22]. We identified patients in nursing homes or residential care at the time of AKI, the annual quarter in which AKI occurred, and, if admitted, the specialty involved during admission with the following priority where more than one was involved: critical care, surgical, care of the elderly, medical and ‘other’ ward (e.g. obstetrics, psychiatry). We collected all comorbidity events in the previous 5 years using International Classification of Diseases (ICD-10) codes for Charlson comorbidities (see Table 2 for list) as previously described and validated [23, 24]. The four-variable Modification of Diet in Renal Disease (MDRD) eGFR is currently reported in the health region, and we used this to describe baseline renal function in four eGFR groups: normal ≥60, mild 45–59, moderate 30–44 and severe impairment <30 mL/min/1.73 m^2^.

**Table 2.** Cohort characteristics by AKI group

	HA-AKI	CAA-AKI	CANA-AKI
*n*	2779	1042	729
Median age in years (IQR)	77 (68–84)	73 (61–82)	72 (60–79)
Age ≥70 years	1994 (71.8)^a^	606 (58.2)	408 (56.0)
Female	1455 (52.4)	542 (52.0)	465 (63.8)
Baseline eGFR group (mL/min/1.73 m^2^)			
≥60	1649 (59.3)	681 (65.4)	451 (61.9)
45–59	527 (19.0)	196 (18.8)	129 (17.7)
30–44	391 (14.1)	117 (11.2)	104 (14.3)
0–29	212 (7.6)	48 (4.6)	45 (6.2)
Year quarter			
January–March	807 (29.0)	321 (30.8)	215 (29.5)
April–June	686 (24.7)	259 (24.9)	173 (23.7)
July–September	608 (21.9)	222 (21.3)	182 (25.0)
October–December	678 (24.4)	240 (23.0)	159 (21.8)
Specialty (if admitted)			
Medicine	879 (31.6)	499 (47.9)	
Elderly care	425 (15.3)	160 (15.4)	
Surgical	466 (16.8)	194 (18.6)	
Critical care	787 (28.3)	148 (14.2)	
Other	222 (8.0)	41 (3.9)	
Patient location			
Residential care	119 (4.3)	92 (8.8)	22 (3.0)
Deprived area (SIMD)	255 (9.2)	99 (9.5)	48 (6.6)
Rural location	752 (27.1)	252 (24.2)	223 (30.6)
Charlson comorbidities from admissions in previous 5 years^b^			
Myocardial infarction	578 (20.8)	77 (7.4)	62 (8.5)
Cardiac failure	601 (21.6)	109 (10.5)	107 (14.7)
Peripheral vascular disease	326 (11.7)	81 (7.8)	37 (5.1)
Cerebrovascular disease	444 (16.0)	81 (7.8)	42 (5.8)
Dementia	186 (6.7)	44 (4.2)	16 (2.2)
Chronic pulmonary disease	445 (16.0)	140 (13.4)	52 (7.1)
Rheumatic	134 (4.8)	37 (3.6)	39 (5.3)
Peptic ulcer	153 (5.5)	47 (4.5)	27 (3.7)
Liver disease—mild	95 (3.4)	27 (2.6)	20 (2.7)
Liver disease—severe	50 (1.8)	11 (1.1)	11 (1.5)
Diabetes—uncomplicated	432 (15.5)	156 (15.0)	90 (12.3)
Diabetes—complicated	80 (2.9)	33 (3.2)	9 (1.2)
Hemiplegia	63 (2.3)	13 (1.2)	6 (0.8)
Malignancy	549 (19.8)	214 (20.5)	95 (13.0)
Metastatic malignancy	177 (6.4)	77 (7.4)	23 (3.2)
Any vascular disease^c^	1542 (55.5)	370 (35.5)	228 (31.3)
0 diseases	573 (20.6)	398 (38.2)	371 (50.9)
1 disease	894 (32.2)	324 (31.1)	174 (23.9)
2 diseases	751 (27.0)	191 (18.3)	118 (16.2)
3 or more diseases	561 (20.2)	129 (12.4)	66 (9.0)

AKI, acute kidney injury; eGFR, estimated glomerular filtration rate; IQR, interquartile range; HA-AKI, hospital AKI; CAA-AKI, community AKI admitted within 7 days; CANA-AKI, community AKI not admitted within 7 days; SIMD, Scottish Index of Multiple Deprivation.

^a^Percentage of group unless otherwise specified.

^b^Human immunodeficiency virus data suppressed to avoid identification.

^c^Vascular disease includes peripheral vascular disease, diabetes, cerebrovascular disease, myocardial infarction and cardiac failure.

### Analysis

We described patient characteristics and the AKI e-alert criteria performance in each of the three AKI groups. Previous studies have separated hospital and community AKI based on different time cut-offs for AKI presentation (e.g. AKI on the day of admission only or AKI up to 48 h after hospital admission) [6, 9]. Therefore, we also reported the number of patients newly meeting AKI criteria on each admission day and their corresponding 30-day mortality. In addition, as our cohort included a 20% sample of patients with normal results, we checked in a sensitivity analysis that the proportions in each AKI group were similar after multiplying out the sampled fraction.

We compared 30-day, and 1- and 5-year mortality between AKI groups and plotted Kaplan–Meier survival curves first unadjusted, then limited to 30-day survivors, and then limited to 30-day survivors and adjusted for age, sex, baseline eGFR and all Charlson comorbidities (listed in Table 2). We used Cox proportional hazards regression to determine the unadjusted and adjusted risk of death for community-acquired AKI admitted and not admitted relative to hospital-acquired AKI both in the short term (30 days) and in the long term (subsequent 5 years in 30-day survivors). We checked the proportional hazards assumption using log–log survival plots. Continuous variables (age, baseline eGFR) were tested with linear, quadratic terms and in categories to determine whether the linear term was adequate. As a sensitivity analysis to provide additional context for AKI outcomes, we also reported 5-year mortality for all people admitted to hospital in 2003, with and without renal impairment at hospital admission (eGFR < or ≥60 mL/min/1.73 m^2^), who did not experience AKI in 2003.

Between AKI groups, we compared recovery status at 90 days, the number of patients who received repeat blood tests within 7 and 90 days and the number of patients receiving chronic RRT at 1 and 5 years. Analysis was performed using Stata/SE 13.0 (StataCorp).

## RESULTS

### Cohort characteristics

Of 50 835 patients, 4550 (9.0%) had AKI (Figure 1). The majority (61.1%) were HA-AKI, but a substantial proportion occurred in the community (22.9% CAA-AKI and 16.0% CANA-AKI). Those with HA-AKI were older, received more critical care and had more comorbidities (Table 2).

### Performance of AKI criteria

We compared each AKI group to determine whether differences in the testing pattern between community and hospitalized patients impacted on how the AKI criteria operated (Table 3). Compared with CAA-AKI and CANA-AKI, those with HA-AKI had more prior tests (median number 7 versus 4 and 5, respectively) and more recent tests (median days between AKI and last test 1 versus 52 and 69). Accordingly, there was substantial difference in how the AKI criteria operated. Those with HA-AKI more frequently met AKI Criteria 2 and 3 (change from prior 7 days), whereas those with CAA-AKI and CANA-AKI more frequently met AKI Criterion 1 (change from prior 8–365 days). Those with CAA-AKI also experienced the greatest creatinine rises (based on AKI stage and doubling of creatinine).

**Table 3.** Performance of AKI criteria by AKI group

	HA-AKI	CAA-AKI	CANA-AKI
	*n* (%)^a^	*n* (%)^a^	*n* (%)^a^
*n*	2779	1042	729
AKI criteria met at first presentation			
Criterion 1 (8–365 days)	1209 (43.5)^a^	968 (92.9)	685 (94.0)
Criterion 2 (7 days)	1246 (44.8)	111 (10.7)	47 (6.4)
Criterion 3 (48 h)	1624 (58.4)	50 (4.8)	31 (4.3)
Criterion 1 met only	606 (21.8)	901 (86.5)	661 (90.7)
Median number of tests in previous year (IQR)	7 (3–13)	4 (2–11)	5 (2–11)
Median days since last prior test (IQR)	1 (1–3)	52 (14–140)	69 (22–168)
Initial AKI stage at first detection			
1	2337 (84.1)	688 (66.0)	624 (85.6)
2	316 (11.4)	211 (20.2)	76 (10.4)
3	126 (4.5)	143 (13.7)	29 (4.0)
Peak AKI stage over 90 days			
1	1868 (67.2)	554 (53.2)	564 (77.4)
2	574 (20.7)	272 (26.1)	97 (13.3)
3	337 (12.1)	216 (20.7)	68 (9.3)
Doubling of creatinine	900 (32.4)	480 (46.1)	155 (21.3)

AKI, acute kidney injury; eGFR, estimated glomerular filtration rate; IQR, interquartile range; HA-AKI, hospital AKI; CAA-AKI, community AKI admitted within 7 days; CANA-AKI, community AKI not admitted within 7 days.

^a^Percentages reported for each group unless otherwise specified.

### Day of AKI detection

**FIGURE 2:**
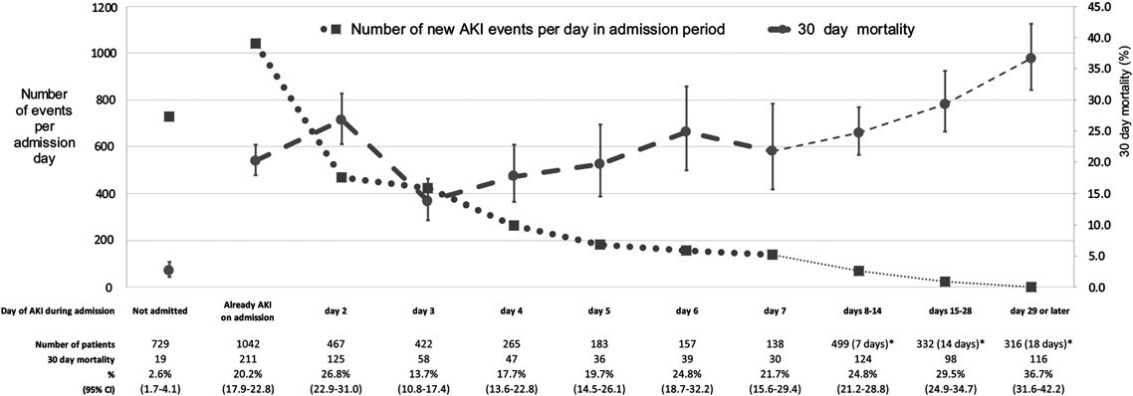
Number of patients with newly detected acute kidney injury (AKI) each day of admission and subsequent 30-day mortality (%). *Number of days in brackets represents as a denominator the median of number of days exposed in the group during that admission period. Error bars represent 95% confidence intervals (CI).

As previous studies separated hospital and community AKI at different time points, we assessed whether this would impact on the proportion of AKI attributed to the community and the subsequent mortality (Figure 2). A total of 38.9% of all AKI events presented in the community or on the day of admission. Alternative definitions would have led to a larger proportion with community AKI (10.2 and 9.3% developed AKI on admission days 2 and 3, respectively).

### Outcomes—mortality

**FIGURE 3:**
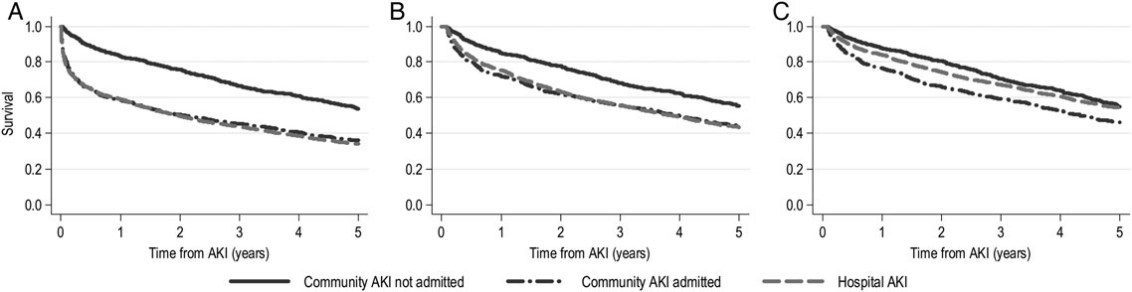
Kaplan–Meier survival in patients with HA-AKI, CAA-AKI and CANA-AKI (**A**) unadjusted; (**B**) limited to 30-day survivors; (**C**) adjusted (for age, baseline eGFR and all Charlson comorbidities as in Table 5) and limited to 30-day survivors. CAA-AKI, community AKI admitted within 7 days; CANA-AKI, community AKI not admitted within 7 days; HA-AKI, hospital acquired AKI. Note that survival curves in (B) and (C) start to fall from 0 years + 30 days. Mortality for CANA-AKI was significantly reduced in (B) but not in (C), as reported in Table 5.

Thirty-day mortality was similar for HA-AKI and CAA-AKI, but lower for CANA-AKI (respectively, 24.2, 20.2 and 2.6%) (Table 4). At 5 years, mortality was substantial in all groups but still lower for CANA-AKI (67.1, 64.7 and 46.2%). However, as shown in the Figure 3 survival curves, the mortality difference (Figure 3A) was attenuated by excluding patients who died within 30 days (Figure 3B) and adjusting for age and comorbidities (Figure 3C). To provide a context, we also determined 5-year mortality for those admitted to hospital who did not have AKI. Five-year mortality was 41.3% in those with baseline renal impairment (*n* = 6486) and 20.7% in those with normal baseline function (*n* = 7584).

**Table 4.** Outcomes in hospital and community AKI

	HA-AKI	CAA-AKI		CANA-AKI	
	*n* (%)	*n* (%)	P-value*	*n* (%)	P-value*
*n*	2779	1042		729	
30-day mortality	673 (24.2)	211 (20.2)	0.010	19 (2.6)	<0.001
1-year mortality	1192 (42.9)	441 (42.3)	0.751	124 (17.0)	<0.001
5-year mortality	1864 (67.1)	674 (64.7)	0.163	337 (46.2)	<0.001
1-year chronic RRT	27 (1.0)	5 (0.5)	0.137	20 (2.7)	<0.001
5-year chronic RRT	44 (1.6)	12 (1.2)	0.323	27 (3.7)	<0.001
Median days to chronic RRT among those who received chronic RRT (IQR)	204 (77–468)	490 (49–845)	0.346	192 (59–482)	0.943
Recovery status at 90 days					
Dead	883 (31.8)	318 (30.5)		46 (6.3)	
Chronic RRT	13 (0.5)	2 (0.2)		9 (1.2)	
Untested	203 (7.3)	102 (9.8)		209 (28.7)	
No recovery	94 (3.4)	36 (3.5)		86 (11.8)	
Partial recovery	316 (11.4)	71 (6.8)		128 (17.6)	
Full recovery	1270 (45.7)	513 (49.2)	<0.001	251 (34.4)	<0.001
Repeat testing (among those alive and without RRT)					
No repeat tests at 7 days	444 (18.6)	187 (20.4)	0.251	593 (81.7)	<0.001
No repeat tests at 90 days	203 (10.8)	102 (14.1)	0.017	209 (31.0)	<0.001

AKI, acute kidney injury; RRT, renal replacement therapy; IQR, interquartile range; HA-AKI, hospital AKI; CAA-AKI, community AKI admitted within 7 days; CANA-AKI, community AKI not admitted within 7 days.

*P-values versus HA-AKI, *χ*^2^ test for comparing proportions, Wilcoxon rank-sum for non-parametric comparison.

In multivariate analysis (reference group HA-AKI), 30-day mortality was similar for CAA-AKI versus HA-AKI (adjusted hazard ratio, HR 1.06, 95% confidence interval, CI 0.89–1.25), but lower in CANA-AKI (HR 0.13, 95% CI 0.07–0.18) (Table 5). Among those still alive at 30 days, adjusted 5-year mortality in CANA-AKI was not significantly lower versus HA-AKI (HR 0.91, 95% CI 0.80–1.04). These results were similar when the analysis was restricted to only those with a doubling of serum creatinine. In summary, mortality was similar for HA-AKI and CAA-AKI. For CANA-AKI, while short-term mortality was low, the long-term mortality was substantial and not statistically significantly different after adjustment.

**Table 5.** Thirty-day mortality and 5-year mortality (in 30-day survivors) by AKI group

	HR (95% CI) for short-term mortality (within 30 days)			HR (95% CI) for long-term mortality (in 30-day survivors)		
	Unadjusted	Age–sex adjusted	Comorbidity adjusted^a^	Unadjusted	Age–sex adjusted	Comorbidity adjusted^a^
HA-AKI	1.00 (ref)	1.00 (ref)	1.00 (ref)	1.00 (ref)	1.0 (ref)	1.00 (ref)
CAA-AKI	0.86 (0.73–1.01)	0.95 (0.81–1.12)	1.06 (0.89–1.25)	1.00 (0.90–1.11)	1.16 (1.04–1.29)	1.21 (1.09–1.35)
CANA-AKI	0.10 (0.06–0.16)	0.11 (0.07–0.18)	0.13 (0.08–0.22)	0.68 (0.60–0.77)	0.83 (0.74–0.94)	0.91 (0.80–1.04)
HA-AKI and doubling of creatinine^b^	1.00 (ref)	1.00 (ref)	1.00 (ref)	1.00 (ref)	1.00 (ref)	1.00 (ref)
CAA-AKI and doubling of creatinine^b^	0.73 (0.59–0.90)	0.78 (0.63–0.96)	0.93 (0.74–1.16)	0.99 (0.84–1.18)	1.08 (0.90–1.28)	1.15 (0.96–1.38)
CANA-AKI and doubling of creatinine^b^	0.16 (0.09–0.31)	0.19 (0.10–0.35)	0.20 (0.11–0.38)	0.76 (0.59–0.98)	0.94 (0.72–1.21)	1.03 (0.78–1.35)

AKI, acute kidney injury; HR, hazard ratio; CI, confidence interval; HA-AKI, hospital AKI; CAA-AKI, community AKI admitted within 7 days; CANA-AKI, community AKI not admitted within 7 days.

^a^Adjustment includes age, sex, baseline eGFR, history of myocardial infarction, cardiac failure, peripheral vascular disease, cerebrovascular disease, dementia, chronic pulmonary disease, rheumatic disease, mild chronic liver disease, severe chronic liver disease, diabetes mellitus with complications, diabetes mellitus without complications, hemiplegia, malignancy, metastatic malignancy and human immunodeficiency virus.

^b^Doubling of serum creatinine during AKI episode used as an alternative definition of AKI excluding those with mild creatinine rises.

### Outcomes—recovery, repeat tests and chronic RRT

As reported in Table 4, non-recovery at 90 days was most common in CANA-AKI. CANA-AKI patients also frequently had no repeat test within 7 days (81.3%) and 90 days (31.0%) of AKI onset. At 5 years, a higher percentage of CANA-AKI patients had initiated chronic RRT than HA-AKI or CAA-AKI (3.7 versus 1.6 and 1.2%, respectively). In further analysis of those who initiated RRT, the median number of days since last test prior to the AKI episode was not prolonged in any group (33 versus 1 and 14 days, respectively), suggesting that misclassification of slow CKD progression would not account for the increased RRT in CANA-AKI, although rapid progression could still be an explanation.

## DISCUSSION

Despite AKI frequently initiating in the community, and despite the need for early recognition of AKI, this is the first large population-based study to explore the implications of applying the same systematic AKI criteria to patients both admitted and not admitted within 7 days. Using NHS England AKI ‘e-alert’ criteria, we report that a substantial proportion of AKI originates in the community, but that AKI criteria operate differently in the community due to less frequent testing. Nevertheless, in those admitted, HA-AKI and CAA-AKI had similar outcomes despite differing antecedent circumstances and baseline characteristics. Notably, 16% of all AKI was not associated with admission within 7 days (CANA-AKI). In this group, the short-term mortality was low, but long-term mortality was substantial.

The high rate of chronic RRT (1 in 30) and lack of repeat testing in CANA-AKI was unexpected. One explanation could be misclassification of rapidly progressing CKD patients when the AKI criteria are applied outside the hospital setting. The low 30-day mortality might also suggest a less ‘acute’ insult in CANA-AKI. However, the creatinine changes were recent even among those not admitted (median 33 days) and this suggests rapid deterioration. Thus, even if AKI criteria misclassify some ‘CKD’ patients in the community, they do still identify a group of patients with significant recent kidney function change, and the lack of repeat testing in this group is therefore still a concern.

This analysis is consistent with previous studies. We confirm that a high proportion of AKI in hospital first originates in the community. While two previous studies reported an even higher proportion [6, 9], this can be explained by the inclusion of patients developing AKI up to 48 h after admission in one study (many of which we classified as HA-AKI) [9], and the assumption of normal baseline renal function if baseline was missing in the other study [6], which could lead to some CKD patients admitted from the community being misclassified as AKI [25]. Our analysis adds greater detail by revealing that AKI criteria perform differently in patients who develop AKI in the community, and that hospital-based studies overlook a sizable group of patients who meet the same AKI criteria, but are not admitted within 7 days. Our short-term mortality findings among HA-AKI (24.2%) and CAA-AKI (20.2%) were also similar to previous reports (28.9 and 20.6%, respectively) [6]. Our analysis goes further by reporting a lower short-term mortality among those not admitted (2.6%), and a high incidence of poor long-term outcomes in all groups irrespective of clinical setting.

This analysis has several strengths: its large size, the use of an unselected population and the availability of all blood tests in a single integrated biochemistry service, minimizing the loss of important baseline data. The use of the widely adopted NHS ‘e-alert’ criteria also makes the findings relevant for practising clinicians.

A relative limitation is our dependence on blood results alone, when the diagnosis of AKI is clinical [3]. We did not include clinical verification, or measures of urine output in our criteria for AKI or proteinuria for baseline CKD. Without clinical verification, patients with baseline CKD can be misclassified due to either repeat sampling variability or infrequent testing [12, 26]. We identified clear differences in how the same criteria functioned when applied in different clinical contexts, and indeed, this is one of the main findings of our analysis. Nevertheless, regardless of the antecedent circumstances, we have shown that pragmatic application of KDIGO-based AKI criteria in the community still identifies clinically relevant patients and in sensitivity analysis we showed that our mortality findings were similar when those with milder creatinine rises (less than double) were excluded. Another limitation is the study year (2003). While this enabled a study of long-term outcomes, recent UK initiatives to raise the awareness of AKI may lead to improvements in monitoring, and the impact of these initiatives should be explored in the future [27]. Also, we described only isolated AKI episodes. Future work should describe the scale and impact of recurrent AKI events on outcomes in community patients, preferably with a non-AKI community comparator group. Future work should also explore whether there are differences in prescribing patterns (e.g. stopping and restarting drugs) in response to AKI in each of these clinical settings.

Our analysis contains two key messages for clinicians. First, many patients first met AKI criteria while still in the community, which suggests that strategies to improve AKI recognition should involve the community. We recognize that responding to community blood tests is challenging as many results return outside of working hours and AKI can occur unexpectedly. The sizable short-term mortality difference between community AKI patients who were and were not admitted within 7 days demonstrates the importance of good clinical judgement (rather than a reliance on blood tests) to prioritize acutely ill patients and avoid unnecessary hospital admissions [28]. Second, regardless of how they operated, AKI criteria identified relevant patients in all settings. AKI criteria may operate differently in the community, but these patients still merit proactive reassessment to confirm or understand why creatinine changes have occurred and manage future risks to avoid recurrence and long-term complications.

Overall, the pragmatic application of the KDIGO AKI criteria in the form of e-alerts results in different performance in different clinical settings, but they still identify patients at risk of poor outcomes. Those who are not admitted may have low short-term mortality, but non-recovery, chronic RRT and long-term mortality are nevertheless high. Thus, patients meeting AKI criteria in the community may not always require hospital admission, but a careful review of the clinical circumstances, preventable risk factors and follow-up is still warranted.

## CONFLICT OF INTEREST STATEMENT

None declared. The results presented in this paper have not been published previously in whole or part.

## References

[1] Bedford M, Stevens P, Wheeler T et al What is the real impact of acute kidney injury? *BMC Nephrol* 2014; 15: 95 24952580 10.1186/1471-2369-15-95 PMC4079645

[2] Chertow GM, Burdick E, Honour M et al Acute kidney injury, mortality, length of stay, and costs in hospitalized patients. *J Am Soc Nephrol* 2005; 16: 3365–3370 16177006 10.1681/ASN.2004090740

[3] Kidney Disease: Improving Global Outcomes (KDIGO) Acute Kidney Injury Work Group. KDIGO clinical practice guideline for acute kidney injury. *Kidney Inter Suppl* 2012; 2: 1–138

[4] Stewart J, Findlay G, Smith N et al *Adding Insult to Injury: A Review of the Care of Patients who Died in Hospital with a Primary Diagnosis of Acute Kidney Injury*. London: National Confidential Inquiry into Patient Outcome and Death, 2009

[5] NHS England. *Patient Safety Alert on Standardising the Early Identification of Acute Kidney Injury, 2014*. http://www.england.nhs.uk/2014/06/09/psa-aki/ (16 October 2014, date last accessed) 10.1159/000439146 26351847

[6] Selby NM, Crowley L, Fluck RJ Use of electronic results reporting to diagnose and monitor AKI in hospitalized patients. *Clin J Am Soc Nephrol* 2012; 7: 533–540 22362062 10.2215/CJN.08970911

[7] James M, Dixon E, Roberts D Improving prevention, early recognition and management of acute kidney injury after major surgery: results of a planning meeting with multidisciplinary stakeholders. *Can J Kidney Health Dis* 2014; 1: 20 25960886 10.1186/s40697-014-0020-y PMC4424428

[8] Wilson FP, Shashaty M, Testani J Automated, electronic alerts for acute kidney injury: a single-blind, parallel-group, randomised controlled trial. *Lancet* 2015; 385: 1966–1974 25726515 10.1016/S0140-6736(15)60266-5 PMC4475457

[9] Wonnacott A, Meran S, Amphlett B et al Epidemiology and outcomes in community-acquired versus hospital-acquired AKI. *Clin J Am Soc Nephrol* 2014; 9: 1007–1014 24677557 10.2215/CJN.07920713 PMC4046725

[10] Schissler MM, Zaidi S, Kumar H et al Characteristics and outcomes in community-acquired versus hospital-acquired acute kidney injury. *Nephrology* 2013; 18: 183–187 23336108 10.1111/nep.12036

[11] Sawhney S, Fluck N, Marks A Acute kidney injury—how does automated detection perform? *Nephrol Dial Transplant* 2015; 30: 1853–1861 25925702 10.1093/ndt/gfv094 PMC4617372

[12] Sawhney S, Marks A, Ali T Maximising acute kidney injury alerts - a cross-sectional comparison with the clinical diagnosis. *PLoS One* 2015; 10: e0131909 26125553 10.1371/journal.pone.0131909 PMC4488369

[13] Hobbs H, Bassett P, Wheeler T Do acute elevations of serum creatinine in primary care engender an increased mortality risk? *BMC Nephrol* 2014; 15: 206 25535396 10.1186/1471-2369-15-206 PMC4289548

[14] National Records of Scotland. *Population Estimates Time Series Data, 2014*. http://www.nrscotland.gov.uk/statistics-and-data/statistics/statistics-by-theme/population/population-estimates/mid-year-population-estimates/population-estimates-time-series-data (17 November 2014, date last accessed)

[15] Marks A, Fluck N, Prescott GJ Looking to the future: predicting renal replacement outcomes in a large community cohort with chronic kidney disease. *Nephrol Dial Transplant* 2015; 30: 1507–1517 25943597 10.1093/ndt/gfv089

[16] University of Aberdeen. *Grampian Laboratory Outcomes Morbidity and Mortality Study (GLOMMS), 2014*. http://www.abdn.ac.uk/iahs/research/chronic-disease/glomms.php (2 December 2014, date last accessed)

[17] Hallan SI, Matsushita K, Sang Y Age and association of kidney measures with mortality and end-stage renal disease. *JAMA* 2012; 308: 2349–2360 23111824 10.1001/jama.2012.16817 PMC3936348

[18] Iqbal S, Clark DN, Morris C *Will the real Joe Bloggs please link up? Assessing the quality of indexing in record linkage*. The Farr International Conference 2015; Abstract 1642. 2015; https://www.eventsforce.net/STANDREWS/media/uploaded/EVSTANDREWS/event_35/Abstract_Book_Master_for_online1708.pdf (11 November 2015, date last accessed)

[19] University of Aberdeen. *Grampian Data Safe Haven, 2014*. http://www.abdn.ac.uk/iahs/facilities/grampian-data-safe-haven.php (2 December 2014, date last accessed)

[20] Talabani B, Zouwail S, Pyart RD et al Epidemiology and outcome of community-acquired acute kidney injury. *Nephrol (Carlton)* 2014; 19: 282–287 10.1111/nep.12221 24571827

[21] UK Renal Registry. UK Renal Registry 17th Annual Report: Appendices. *Nephron* 2015; 129 (Suppl 1): 273–276 10.1159/000370283 25695817

[22] ISD Scotland. *Deprivation and Urban Rural Measurements in ISD, 2004*. http://www.isdscotlandarchive.scot.nhs.uk/isd/3211.html (8 August 2015, date last accessed)

[23] Quan H, Li B, Couris CM Updating and validating the Charlson comorbidity index and score for risk adjustment in hospital discharge abstracts using data from 6 countries. *Am J Epidemiol* 2011; 173: 676–682 21330339 10.1093/aje/kwq433

[24] Johnston MC, Marks A, Crilly MA et al Charlson index scores from administrative data and case-note review compared favourably in a renal disease cohort. *Eur J Public Health* 2015; 25: 391–396 25583040 10.1093/eurpub/cku238

[25] Siew ED, Peterson JF, Eden SK et al Use of multiple imputation method to improve estimation of missing baseline serum creatinine in acute kidney injury research. *Clin J Am Soc Nephrol* 2013; 8: 10–18 23037980 10.2215/CJN.00200112 PMC3531649

[26] Lin J, Fernandez H, Shashaty MGS False-positive rate of AKI using consensus creatinine–based criteria. *Clin J Am Soc Nephrol* 2015; 10: 1723–1731 26336912 10.2215/CJN.02430315 PMC4594067

[27] NHS England. *Acute Kidney Injury (AKI) Programme, 2014*. http://www.england.nhs.uk/ourwork/patientsafety/akiprogramme/ (16 October 2014, date last accessed)

[28] Blakeman T, Harding S, O'Donoghue D Acute kidney injury in the community: why primary care has an important role. *Br J Gen Pract* 2013; 63: 173–174 23540451 10.3399/bjgp13X664207 PMC3609441

